# Management of Bilateral Subacute Subdural Hematomas with Concomitant Subarachnoid Hemorrhage in a Diabetic Patient with HIV in Cameroon: A Case Report

**DOI:** 10.7759/cureus.115967

**Published:** 2026-09-08

**Authors:** Mireille Georgette Moumi, Philippe-Albert Lingo, Joseph Fondop, Junette Arlette Metogo Mbengono, Vincent de Paul Djientcheu

**Affiliations:** 1 Department of Surgery and Specialties, Anesthesia and Intensive Care, Faculty of Medicine and Pharmaceutical Sciences, University of Dschang, Dschang, CMR; 2 Department of Internal Medicine and Pediatrics, Faculty of Health Sciences, University of Buea, Buea, CMR; 3 Department of Anesthesia and Critical Care, Faculty of Medicine and Pharmaceutical Sciences, University of Douala, Douala, CMR; 4 Department of Surgery and Specialties, Faculty of Medicine and Biomedical Sciences, University of Yaounde I, Yaounde, CMR

**Keywords:** bilateral subacute subdural hematomas, cameroon, craniotomy, diabetic adult, hiv, subarachnoid hemorrhage

## Abstract

Bilateral subdural hematomas occurring simultaneously with subarachnoid hemorrhage in a traumatic context are rare and pose considerable diagnostic challenges. Comorbidities such as diabetes and HIV may further worsen prognosis and complicate management. This case underscores the vital importance of early diagnosis and multidisciplinary care in patients with subacute subdural hematomas and subarachnoid hemorrhage, particularly when compounded by comorbidities in resource‑limited settings.

We report the case of a 44-year-old housewife who was diabetic and living with HIV. She presented with severe headaches followed by altered consciousness with severe coma, and Cushing’s triad two weeks after a minor head trauma. Brain CT scan revealed a bilateral panhemispheric subacute subdural hematoma (SASDH) associated with subarachnoid hemorrhage. Management consisted of emergent bilateral burr hole craniotomy for drainage, combined with measures to reduce intracranial pressure and prevent secondary brain injury of systemic origin. Her course was complicated by infection and hypertensive crisis; both were successfully managed. Glycemic control was achieved and antiretroviral therapy optimized. Clinical and radiological outcomes were ultimately favorable.

This case underscores the importance of early diagnosis and multidisciplinary management of subdural hematomas in patients with diabetes and HIV. Beyond timely surgical intervention with burr‑hole craniotomy, comprehensive intensive care - including vigilant monitoring, early detection, and prompt control of complications - can significantly improve survival and optimize neurological outcomes.

## Introduction

Subdural hematoma (SDH) is a blood collection located between the dura mater and the arachnoid, most often resulting from the rupture of bridging veins following a cranial trauma, which may be minor or even unnoticed [1]. Based on symptom onset, SDH is classified as acute (≤three days), subacute (three to 21 days), or chronic (>21 days). Subacute SDH (SASDH) represents an intermediate entity, frequently diagnosed late due to its progressive and nonspecific presentation [2].

Concerning epidemiology, Robinson et al. found an overall incidence of 17.3 per 100,000 persons per year after adjustment to national demographics, with a steep age gradient reaching 354.8 per 100,000 in men aged 85 years or older [3]. Meanwhile, in Cameroon, SDHs constituted 39.7% of traumatic intracranial hemorrhages, with diabetes being the second most common bleeding risk factor associated with it [4]. The simultaneous occurrence of bilateral SDH and subarachnoid hemorrhage (SAH) remains relatively rare and presents particular diagnostic and therapeutic challenges, especially in the absence of obvious head trauma. Comorbidities such as diabetes mellitus and infection with the human immunodeficiency virus (HIV) may promote coagulopathy and vascular fragility [5], as well as sepsis or central nervous system (CNS) infections in the postoperative period [6], thereby influencing both the presentation and increasing the complexity of management. Depending on the timing of onset, SDHs may present with headaches, vomiting, altered consciousness, seizures, or focal neurological deficits. These manifestations are nonspecific; therefore, differential diagnoses such as intracranial abscess, hemorrhagic stroke, and intracranial neoplasm must be considered. The first‑line diagnostic tool remains non‑contrast head CT, which typically reveals crescent‑shaped collections that cross suture lines but are limited by the falx and tentorium. Acute complications include secondary cerebral injury, herniation, and seizures, while postoperative complications may involve persistent mass effect, rebleeding, hydrocephalus, and infection [7]. Emergency surgical intervention, preferably via burr‑hole craniotomy, is indicated in patients with SDH who are symptomatic or demonstrate significant mass effect on imaging. Conversely, conservative management may be appropriate in cases with mild clinical presentation and limited mass effect [8].

We present the case of a 44-year-old woman presenting with this association following minor head trauma. This case highlights the clinical, radiological and therapeutic, as well as the emergency management challenges in resource‑limited settings, with the aim of edifying on the strategies to optimize neurological outcomes.

## Case presentation

A 44‑year‑old woman with type 2 diabetes and HIV, adherent to treatment, was admitted unconscious after transfer from another facility. She had sustained a mild head injury in a road traffic accident three weeks earlier. Two weeks later, she developed progressively worsening headaches unresponsive to analgesics, followed by altered consciousness. Non‑contrast brain CT revealed bilateral subacute SDHs with mass effect (Figure 1) and associated SAH (Figure 2).

**Figure 1:**
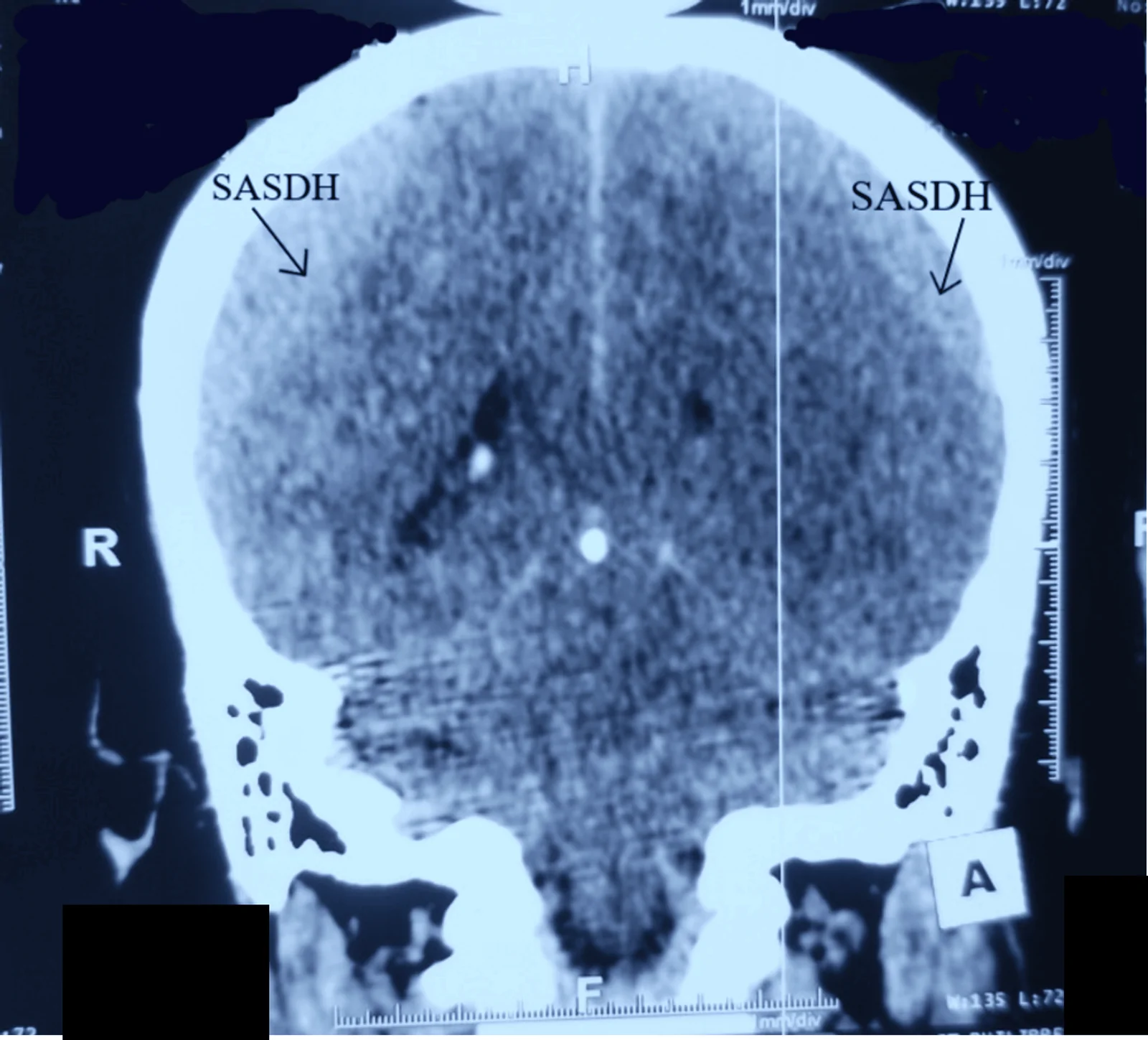
Frontal view of the brain CT Shows bilateral parietal crescent-shaped hyperdense collections along both parietal regions suggesting subacute subdural hematoma (SASDH) with mass effect on adjacent structures

**Figure 2:**
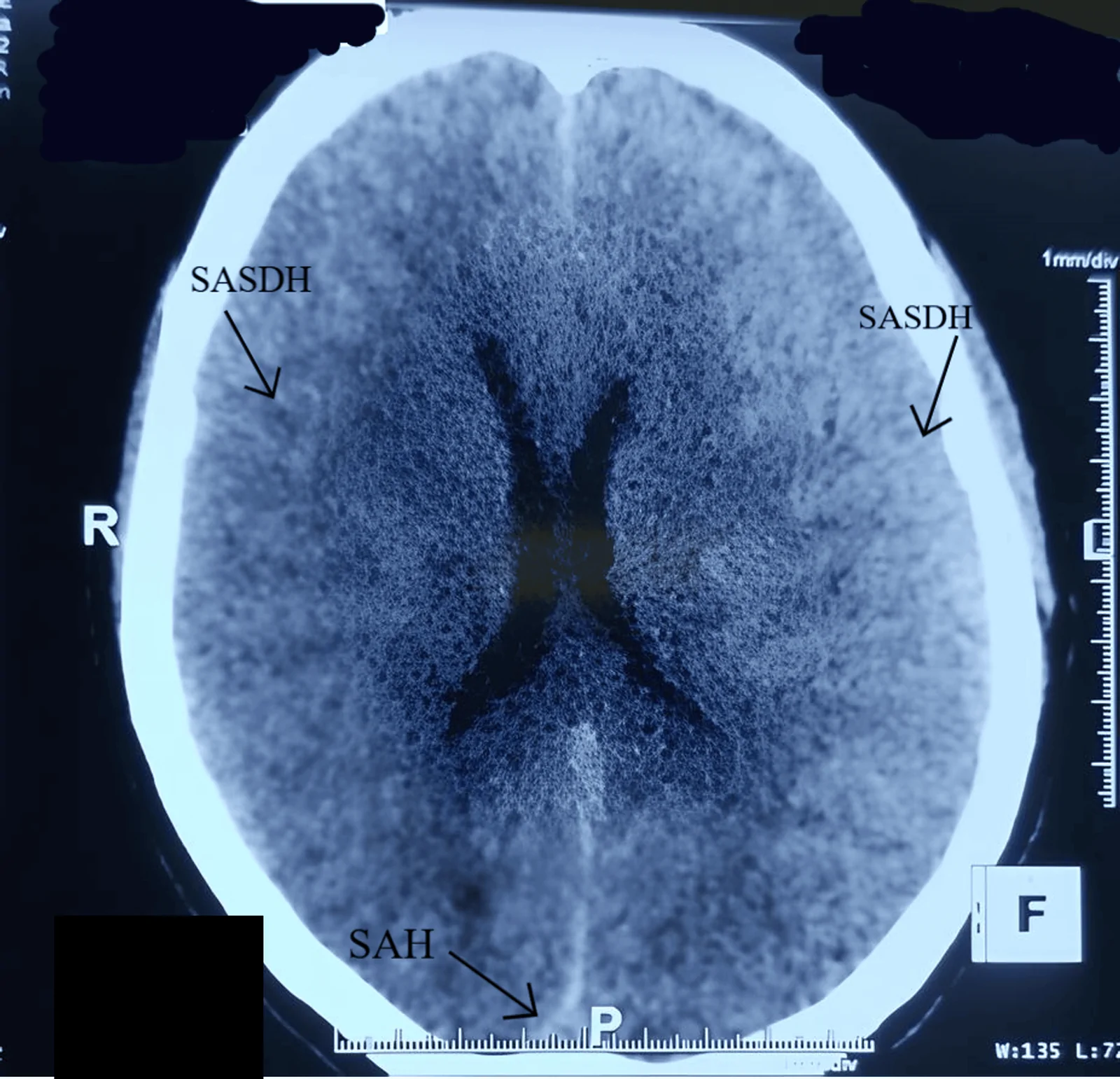
Axial view of the brain CT Shows hyperdensity in the sulci and basal cistern suggesting subarachnoid hemorrhage (SAH) and bilateral collection of subacute subdural hematoma (SASDH).

The latter findings motivated the referral to our facility for better management.

On admission, Glasgow Coma Scale was 6/15 with reactive bilateral mydriasis and Cushing’s triad (hypertension 222/118 mmHg (reference value <120/80 mmHg), bradycardia 55 bpm (reference value 60-100 bpm), irregular respiration (reference value 12-20 breaths per minute)), consistent with severe intracranial hypertension. Signs of meningeal irritation were also noted. The remainder of physical exam was normal. Oxygen saturation was 90% on room air; capillary glucose was 180 mg/dl. Laboratory values, including coagulation and platelets, were normal. The results of laboratory investigations are shown in Table 1.

**Table 1:** Results of laboratory investigations

Laboratory test	Result	Normal values
White blood cells	9000/mm^3^	4000-10000/mm^3^
Hemoglobin	12 g/dL	11-18 g/dL
Platelets	151000/mm^3^	150000-300000/mm^3^
Prothrombin Time	11 seconds	10-13.5 seconds
Activated Partial Thrombin Time	27 seconds	25-35 seconds
Urea	50 mg/dL	10-50 mg/dL
Creatinine	0.9 mg/dL	0.7-1.1 mg/dL
Sodium	137 mEq/L	135-155 mEq/L
Potassium	4.0 mEq/L	3.4-5.3 mEq/L
Chloride	109 mEq/L	90-110 mEq/L
Blood glucose	180 mg/dL	60-110 mg/dL

She was immediately intubated, ventilated, and started on intravenous nicardipine, mannitol, analgesics, and nimodipine. Emergent bilateral burr‑hole craniotomy evacuated 60 ml of pressurized blood per side, followed by irrigation and placement of bilateral subdural drains. These procedures were carried out after counselling was provided to the family regarding the severity of the clinical condition requiring emergency surgery. Postoperatively, she remained ventilated and received ceftriaxone, nimodipine, and supportive care.

Postoperative course was complicated by grade 3 hypertension requiring IV labetalol, hyperglycemia managed with insulin infusion, and fever with leukocytosis and elevated CRP. Antibiotics were escalated to meropenem and amikacin; prophylactic anticoagulation, physiotherapy, and pressure ulcer prevention were instituted. The results of some of the pertinent investigations done are shown in Table 2. 

**Table 2:** Laboratory findings on postoperative day five

Biological parameter	Patient's value	Normal reference range	Interpretation
White blood cell count (WBC)	17800/mm^3^	4000-10000/mm^3^	Leukocytosis, suggestive of an active infectious/inflammatory process
C-reactive protein (CRP)	217 mg/L	<5 mg/L	Marked systemic inflammatory response, highly suggestive of severe infection
Thick blood smear	Negative	Negative	Malaria excluded
Urine dipstick test	Normal	Normal	No evidence of urinary tract infection
Chest X-ray	Normal	Normal	No radiological evidence of pulmonary infection

On postoperative day five, extubation was difficult due to the patient's inability to tolerate spontaneous ventilation. A control brain CT scan was done on postoperative day five (Figure 3).

**Figure 3:**
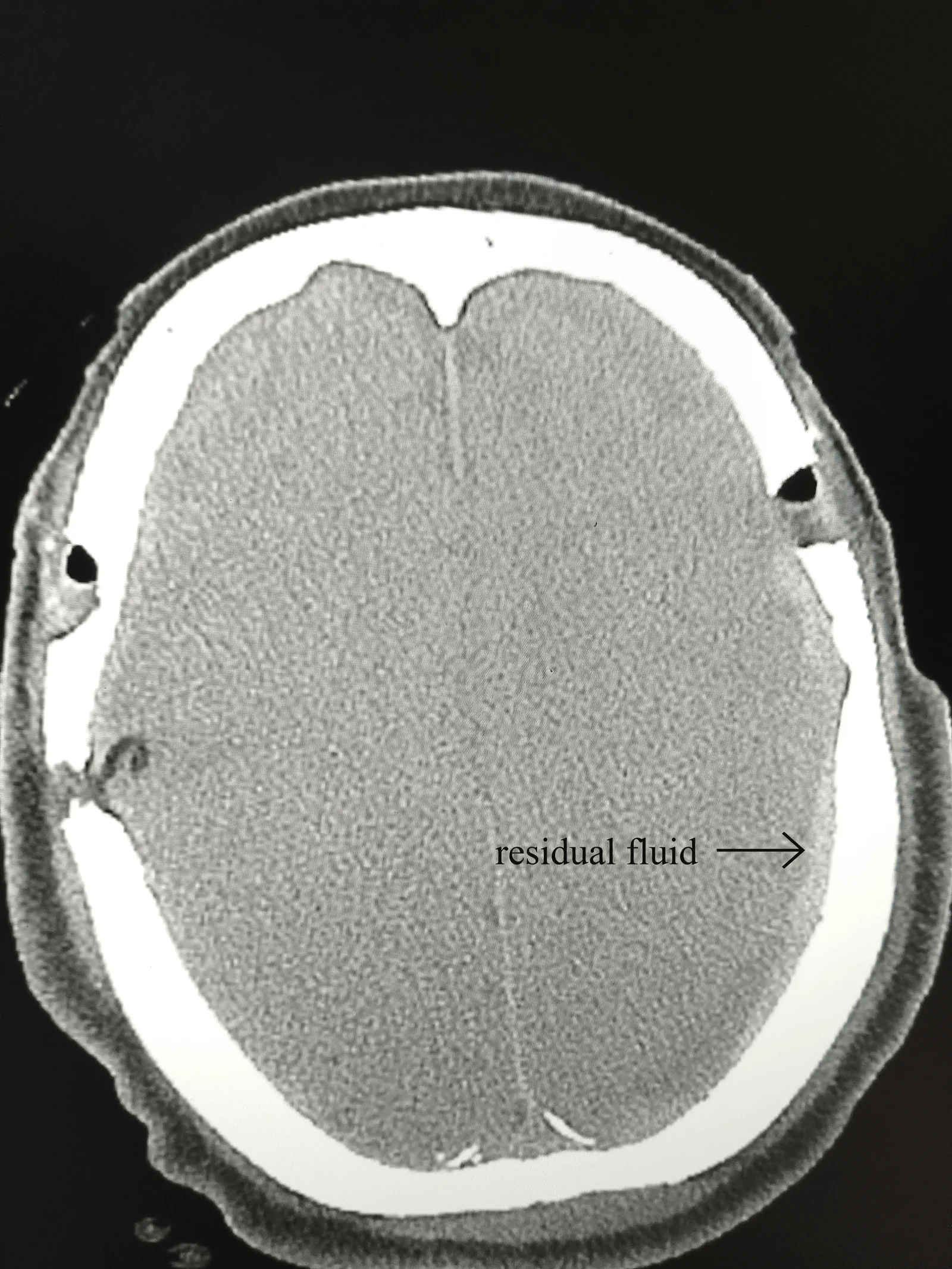
Control brain CT scan on postoperative day five showing considerable regression of the subdural collections with presence of residual fluid

A probable early encephalitis related to a possible surgical entry point could not be excluded; however, lumbar puncture was contraindicated given the patient's elevated intracranial pressure. Moreover, the hyperthermia as well as hypertension and hyperglycemia may represent sources of secondary brain injury of systemic origin.

Neurological improvement allowed progressive weaning from ventilation by day nine. Extubation was achieved on day 11 after stabilization of fever, hypertension, and regression of a stage one pressure ulcer. Glycemic control required ongoing insulin adjustment, and antiretroviral therapy was optimized. On postoperative day 13, the Glasgow score was 13/15, the bilateral mydriasis had reduced, spontaneous ventilation was well tolerated, blood pressure was 140/89 mmHg, and blood glucose was controlled.

By day 15, Glasgow score was 15/15, hemodynamic stability was achieved, and antihypertensives were discontinued. No neurological deficits remained. She was discharged on postoperative day 21 with outpatient neurosurgical, diabetic, and HIV follow‑up, resuming normal daily activities.

The clinical evolution of the patient is summarized in Table 3.

**Table 3:** Evolution of the patient's clinical course

Timeline (Weeks after trauma)	Clinical events & findings
Week 0 (Day of trauma)	Mild head injury following a road traffic accident. No immediate neurological deficits reported.
Week 2	Onset of progressively worsening headaches, resistant to usual analgesics.
Week 3	Altered consciousness leading to coma; Glasgow Coma Scale 6/15, Cushing’s triad (hypertension, bradycardia, irregular respiration). CT scan revealed bilateral subacute subdural hematoma with subarachnoid hemorrhage.
Day 1 of admission	Intubation, mechanical ventilation, bilateral burr hole craniotomy with evacuation of hematoma, subdural drains placed, mannitol infusion, nicardipine for BP control, nimodipine for vasospasm prevention.
Day 5 (postoperative)	Persistent intracranial hypertension, fever, hyperglycemia; antibiotics escalated.
Day 9–11 (postoperative)	Neurological improvement, progressive weaning from ventilation, extubation achieved.
Day 13–15 (postoperative)	Glasgow score improved to 15/15, hemodynamic stability, glycemic control achieved.
Day 21 (postoperative)	Full neurological recovery, transferred for follow‑up of comorbidities (diabetes, HIV).

## Discussion

Co-occurrence of SDH and SAH is rare, with no existing epidemiologic data, although some cases have been reported. For example, Soedjono et al. described a case involving concomitant occurrence of SDH with subarachnoid SAH and intracerebral hemorrhage secondary to a ruptured brain arteriovenous malformation [9], while another report presented a case of acute SDH and SAH due to ruptured cortical artery aneurysm [10]. Both of the latter cases were spontaneous, while our case had a traumatic origin. Notably, our patient presented with symptomatic delayed bilateral SASDH, which developed two weeks after an initially mild traumatic brain injury following a road traffic crash. This warrants careful monitoring of patients who initially sustain mild head trauma, especially in the context of a road traffic crash. This patient also showed CT findings suggestive of SAH. This could also be explained by the traumatic event [11]. Our patient had diabetes mellitus and HIV, both comorbidities that play a role in the development and progression of SDHs. Diabetes mellitus increases the risk of SDH by promoting microvascular disorders and coagulopathies, thereby worsening the prognosis of intracranial lesions [12]. Similarly, an HIV infection is linked to inflammation leading to endothelial dysfunction and hematologic disorders, all of which may predispose to SDHs [13].

The SASDH and SAH in this patient were diagnosed by non-contrast brain CT, which is the gold standard imaging modality for traumatic brain injuries [14]. Nevertheless, it would have been of benefit to have a cerebral CT angiography performed for this patient given the finding suggestive of SAH, but due to her critical state and the need for urgent intervention, this was not feasible.

Given the deep comatose state of the patient with a Glasgow Coma Score of 6/15, endotracheal intubation had to be done, and the patient was placed on mechanical ventilation following this indication [15]. The bilateral SASDH was associated with elevated intracranial pressure. Thus, emergency surgical evacuation of the hematoma was done through bilateral burr-hole craniotomy [16]. This approach is similar to the management strategy reported by Nagpal et al. [17]. Concerning the presentation of SASDH, it should be noted that our patient had a more severe clinical presentation with poorer prognosis and a complicated postoperative course as compared to the case reported by Nagpal et al [17]. Furthermore, mannitol boluses were given to reduce cerebral edema and to reduce elevated intracranial pressure [18]. No surgical intervention was done for the SAH. This was probably due to the absence of angiography and the “burr hole” technique used, which could not allow the identification of the site of the SAH for eventual correction as in the cases previously mentioned [9,10].

SAH predisposes to secondary cerebral vasospasms with delayed cerebral ischemia which may worsen the cerebral assault and eventually lead to poorer neurologic outcomes. In order to mitigate this risk, nimodipine was administered in accordance with management guidelines [19]. Since the patient was diabetic, she was at risk of hyperglycemic crisis in the intensive care unit, which she eventually developed. In order to maintain glycemic control, an insulin infusion protocol was instituted with the target values between 110 mg/dl and 180 mg/dl. These targets are consistent with current guidelines which recommend glycemic targets of 140 mg/dl to 180 mg/dl in critically ill patients with brain injury [20]. Given the patient's elevated blood pressure which could increase the risks of intracranial rebleeding, blood pressure had to be controlled with a target of systolic blood pressure between 110 to 160 mmHg [19,21].

The patient’s evolution was favorable with the subsequent review and follow-up, and management of her comorbidities.

## Conclusions

This case illustrates how comorbidities such as diabetes mellitus and HIV can significantly compromise the prognosis of patients with SASDH and SAH, often leading to complex postoperative courses. Optimal care in such settings requires a carefully structured management strategy that integrates timely surgical intervention, appropriate medical therapy, and rigorous monitoring with structured follow‑up. Moreover, it emphasizes the need for heightened vigilance in patients presenting with even minor head injuries and highlights the critical importance of close collaboration among neurosurgeons, neurologists, critical care physicians, and infectious disease specialists to optimize both short‑ and long‑term outcomes.
